# Etymologia: *Pneumocystis jirovecii*

**DOI:** 10.3201/eid2308.ET2308

**Published:** 2017-08

**Authors:** Ronnie Henry

**Keywords:** etymologia, Pneumocystis jiroveci, fungi, pneumonia, Antonio Carini, Otto Jirovec

## *Pneumocystis jirovecii* [nooʺmo-sisʹtis yeʺro-vetʹze] 

A genus of unicellular fungi, *Pneumocystis* (Figure) was likely originally described by Carlos Chagas in 1909 in guinea pigs, although he confused it with a trypanosome and placed it in a new genus, *Schizotrypanum*. In 1912, Delanoë and Delanoë at the Pasteur Institute published the first description of the new organism as unrelated to trypanosomes and proposed the species name *P. carinii* in honor of Antonio Carini.

**Figure:**
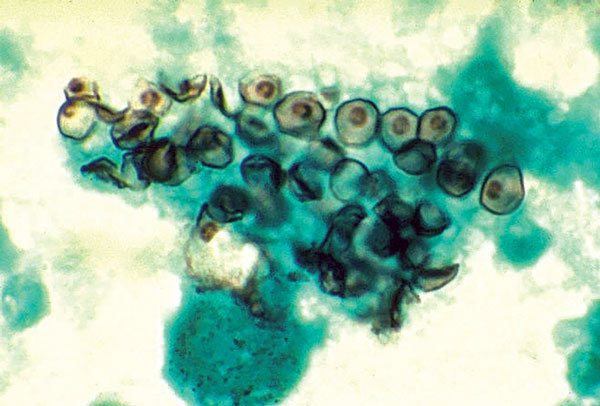
Cysts of Pneumocystis jirovecii in smear from bronchoalveolar lavage. Methenamine silver stain. CDC/Dr. Russell K. Brynes

Human *Pneumocystis* infections were first reported in 1942 by van der Meer and Brug, but not until 1976 did Frenkel report different morphologic and physiologic characteristics of human and rat *Pneumocystis* isolates. He proposed the name *P. jirovecii* in honor of Czech parasitologist Otto Jírovec, who reported *Pneumocystis* as a cause of interstitial pneumonia in infants, although this name change was not accepted by researchers at the time. When *Pneumocystis* was reclassified from a protozoan to a fungus, the naming convention shifted from the International Code of Zoological Nomenclature to the International Code of Botanical Nomenclature, and the species epithet was modified from *jiroveci* to *jirovecii*. 
